# Klinische Qualitätssteuerung – ein praktischer Versuch in der Hausarztpraxis am Beispiel der Influenzaimpfung

**DOI:** 10.1007/s44266-023-00060-0

**Published:** 2023-05-26

**Authors:** Vera Souhrada, Mirjam Zrenner, Emmily Schaubroeck, Marco Roos, Thomas Kühlein

**Affiliations:** 1 MVZ-Eckental, Universitätsklinikum Erlangen, Erlangen, Deutschland; 2 Department of Public Health and Primary Care, Universität Gent, Gent, Belgien; 3 Institut für Allgemeinmedizin, Universitätsklinikum Augsburg, Augsburg, Deutschland; 4 Allgemeinmedizinisches Institut, Universitätsklinikum Erlangen, Universitätsstr. 29, 91054 Erlangen, Deutschland

**Keywords:** Qualitätsmanagement, Allgemeinmedizin, Immunisierung, Priorisierung, Quality management, Family medicine, Immunization, Prioritization

## Abstract

Klinische Qualitätssteuerung (KQS) meint ein Qualitätsmanagement im klinischen Bereich. Vermutlich aufgrund der Coronapandemie meldeten sich im Jahr 2020 deutlich mehr influenzaimpfwillige Patient*innen als in den Vorjahren, sodass sich abzeichnete, dass für Hochrisikopatient*innen nicht ausreichend Impfdosen zur Verfügung stehen würden. Um dem Problem zu begegnen, setzten wir einen KQS-Zyklus in Gang. Bei diesem Artikel handelt es sich explizit nicht um eine wissenschaftliche Arbeit, sondern um die exemplarische Beschreibung einer Priorisierung und einer KQS als Beispiel, zur Anregung und zur Diskussion. Wir erstellten folgenden Prozess: 1. Evaluation des Ist-Zustands; 2. Priorisierung der Patient*innen, die sich als impfwillig gemeldet hatten, und Impfung der Hochrisikogruppe zuerst; 3. Identifikation, telefonische Kontaktierung und Impfung der Hochrisikopatient*innen, die sich nicht selbstständig als impfwillig gemeldet hatten. Patient*innen über 60-Jahre mit chronisch obstruktiver Lungenerkrankung (COPD) wurden als Indikatorgruppe gewählt. Zunächst waren nur 3 (8 %) unserer 38 COPD-Patient*innen gegen Influenza geimpft. Nach Priorisierung und Impfung der Hochrisikogruppe in der Liste der Impfwilligen waren 25 (66 %) der insgesamt 38 COPD-Patient*innen geimpft. Nach telefonischer Einladung und Aufforderung waren zuletzt 28 (74 %) der 38 Patient*innen geimpft. Dies entspricht einer Steigerung der Impfquote der über 60-jährigen COPD-Patient*innen von 8 % auf 74 % und damit nahezu dem Zielwert der Weltgesundheitsorganisation (WHO). Hausärzt*innen müssen sich gelegentlich mit Ressourcenknappheit auseinandersetzen und sich mit Strategien beschäftigen, ihr zu begegnen. Unter anderem in diesem Zusammenhang bietet sich eine KQS in der Hausarztpraxis an. Die Praxisverwaltungssysteme (PVS) sollten für diesen Zweck von Seiten der Hersteller benutzerfreundlicher gestaltet werden.

## Abstract (en)

We understand clinical quality governance (CQG) as quality management in the clinical domain. In 2020, presumably due to the coronavirus pandemic, more patients requested to be vaccinated against influenza as compared to previous years so that it became apparent that there would be a shortage for high-risk patients. To meet the problem, we started a CQG process. This article is explicitly not a research article but an exemplary description of a CQG process intended as a stimulus and for discussion. We initiated the following process: (1) evaluation of the present state, (2) patients who already had requested a vaccination were prioritized and vaccinated first, and (3) contacting via telephone and vaccination of high-risk patients not on the list. We chose patients with chronic obstructive pulmonary disease (COPD) older than 60 years as an indicator for the group of highest priority. In the beginning only 3 (8%) of our 38 patients with COPD were vaccinated against influenza. After prioritization and vaccination of the high-risk collective in the list of those who had requested to be vaccinated, 25 (66%) of our 38 patients with COPD were vaccinated. After a phone call of high-risk patients not on the list, 28 (74%) patients were vaccinated. This represents an increase of vaccination coverage from 8% to 74% which is close to the rate recommended by the World Health Organization (WHO). In times of a pandemic, family physicians occasionally have to deal with a scarcity of resources and have to develop strategies for fair resource allocation. Not only in this context is CQG worth the effort. The generation of list queries could be improved by the providers of electronic patient records.

Hausärztliche Arbeit ist unvermeidlich ein wenig chaotisch [1]. Die Patient*innen kommen in großer Dichte, oft unangemeldet oder eingeschoben, und mit vielen Problemen gleichzeitig. Oft gehen wichtige Dinge unter, Impfungen zum Beispiel. Keiner von uns hat die Organisation von Praxen oder gar die Organisation der klinischen Versorgung je gelernt. Jede macht es so gut sie kann. In diesem Artikel stellen wir unseren bescheidenen Versuch vor, einmal ein Problem systematisch und mithilfe der Praxis-EDV anzugehen. Wir bilden uns nicht ein, den Stein der Weisen gefunden zu haben, sondern fordern dazu auf, hier in der ZFA, eigene Lösungen für dieses und andere Probleme miteinander zu teilen.

Klinische Qualitätssteuerung (KQS) meint das Qualitätsmanagement (QM) medizinischer Prozesse [2, 3]. Dabei geht es nicht etwa darum, alle Patient*innen nach dem gleichen Schema zu behandeln, sondern das im Praxisalltag häufige Vergessen wichtiger klinischer Entscheidungen zu reduzieren sowie für eine sinnvolle Ressourcenallokation zu sorgen.

Seit vielen Jahren gibt es eine Empfehlung der Ständigen Impfkommission (STIKO) für die saisonale Influenzaimpfung für besondere Indikationsgruppen sowie auch für Menschen über 60 Jahre [4]. Die Weltgesundheitsorganisation (WHO) hatte für das Jahr 2010 eine Influenzaimpfquote von 75 % für ältere Menschen und chronisch Kranke als Ziel formuliert [5]. Die deutschen Impfraten für Influenza waren zuletzt unbefriedigend, mit Werten zwischen 24 % und 62 % bei über 60-Jährigen und chronisch Kranken in der Saison 2018/2019 [4, 6]. Bislang zeigten sich viele unserer Patient*innen eher mäßig impfwillig. Häufig äußerten sie Kritik am Impfstoff und den vermeintlichen Nebenwirkungen. Selbst die Hochrisikogruppe der meist über 60-jährigen Patient*innen mit chronisch obstruktiver Lungenerkrankung (COPD) war in unserer Praxis in vorangegangen Jahren nur zu geringem Anteil gegen Influenza geimpft. Im Jahr 2017 lag deren Impfquote nur bei 37 % [7]. In unserer Praxis blieben jedes Jahr sogar Impfdosen übrig.

Dies veränderte sich im Jahr 2020 durch die Coronapandemie grundlegend. Bereits im September mussten unsere medizinischen Fachangestellten viel Zeit darauf verwenden, Anfragen bezüglich der Influenzaimpfung zu bearbeiten. Wir hatten bereits 30 % mehr Impfdosen als in den Vorjahren bestellt und begannen Mitte Oktober mit den Impfungen gemäß den STIKO-Empfehlungen. Da diese weit gefasst sind und insbesondere in diesem Jahr, noch in Ermangelung eines Impfstoffs gegen COVID-19, aufgrund der Pandemiepläne der Bundesländer die Influenzaimpfung medial breit beworben wurde, bemerkten wir nach einigen Tagen, dass sehr viele junge, gesunde Patient*innen die Impfung erhalten hatten und sich die Impfstoffmenge rasch reduzierte. Um den Fokus weiterhin, wie empfohlen, auf die Patient*innen der Risikogruppe legen zu können, sahen wir es zwei Wochen nach Impfstart als dringend notwendig an, einen aktuellen Status der Impfquoten unserer Risikogruppen zu erheben.

Alte, multimorbide Patient*innen suchen die Praxis selten „nur für eine Impfung“ auf. Oft erhielten diese Patient*innengruppen in den Vorjahren im Rahmen anderer Termine, wie Laborkontrollen oder Disease-Management-Programm(DMP)-Termine, opportunistisch die Influenzaimpfung. Obwohl sich in den letzten Jahren die Impfraten bei COPD-Patient*innen in unserem medizinischen Versorgungszentrum (MVZ) nach Einführen einer Checkliste für die Versorgung von Patient*innen mit COPD von 34 % (zum 01.04.2016) auf 55 % (zum 01.01.2019) steigern ließen [7], schien es außerhalb der DMP-Folgekonsultationen weiterhin schwierig, diese Patient*innen zu erreichen. In einem Cochrane- Review aus dem Jahr 2018 erwies sich ein telefonisches Erinnerungssystem als am wirkungsvollsten [8].

Um prüfen zu können, ob unsere subjektive Einschätzung der Versorgungsqualität in unserem Medizinischen Versorgungszentrum (MVZ) mit der dann gefundenen realen Versorgungsqualität übereinstimmt, ließen wir alle Ärzt*innen die aktuelle Impfquote für die Risikopatient*innen schätzen. Die von den Ärztinnen unseres MVZ geschätzte Impfquote der betrachteten Risikogruppe lag vor der Statuserhebung, Anfang November, bei „gefühlten“ 50 %. Wie die Vorerfahrungen mit KQS gezeigt hatten, ist es erste Voraussetzung jeder Qualitätsverbesserung, die aktuelle Versorgungsqualität korrekt darzustellen [7].

Es ergaben sich für uns folgende 3 Fragen:

1. Entspricht die gefühlte Impfquote der Wirklichkeit? Das heißt: Welcher Anteil der Hochrisikopatient*innen der im Sinne eines Indikators für vulnerable Patient*innen gewählten Gruppe der über 60-jährigen COPD-Patient*innen war vor unserer Intervention bereits gegen Influenza geimpft?
2. Wie kann eine Priorisierung der Hochrisikogruppe bei Impfstoffknappheit in der Coronapandemie in der Hausarztpraxis konkret umgesetzt werden?
3. Wie weit lässt sich die Impfquote der priorisierten Patient*innen verbessern, indem diese aktiv kontaktiert werden?

Dieser Artikel hat nicht einen wissenschaftlichen Erkenntnisgewinn im Sinne einer Studie zum Ziel. Es geht uns vielmehr um die Anregung einer vermutlich nicht in allen Praxen üblichen Herangehensweise. Viele Praxen werden ähnliche Ansätze verfolgt haben. Die Darstellung unserer Probleme und Lösungen auf dem Weg zu einer KQS sollen Austausch und Diskussion zu diesem Thema befördern. Unser Vorgehen wird nachfolgend im Einzelnen dargestellt.

## Erstellung einer Patient*innenliste im Praxisverwaltungssystem

Um im Vorfeld die Notwendigkeit und den Erfolg einer Intervention evaluieren zu können, wurde zunächst der Ist-Zustand erhoben. Da unser Praxisverwaltungssystem (PVS) keine komplexeren Abfragen erlaubt, wählten wir im Sinne eines Indikators für alle Hochrisikopatient*innen sowie zur vereinfachten Abfrage und Darstellung die Gruppe der über 60-jährigen COPD-Patient*innen aus. Die Wahl dieses Indikators beruhte auf der Tatsache, dass diese Gruppe zahlenmäßig die größte Untergruppe unter den Risikopatient*innen darstellte. Eigentlich definierten wir jedoch unsere Hochrisikogruppe, die mit höchster Priorität geimpft werden sollte, wie im Folgenden beschrieben, deutlich breiter.

Die Datenerhebung erfolgte in Form der Erstellung einer Patient*innenliste, bei der wir ein entsprechendes Programmmodul unseres PVS (MediStar, CompuGroup Medical, Koblenz, Deutschland) nutzten. Mithilfe der Kombination von Freitextsuche und Zeilentypen konnten so z. B. Patient*innen mit der Diagnose „COPD“, einem Alter über 60 Jahren und mit der Bedingung des Influenzaimpfstatus in jeweils definierten Zeiträumen gefiltert werden. Im Fall unseres PVS sind die Eingaben nach dem Eintrag von „PLIST“ (Kürzel für Patient*innenliste) in der Eingabezeile wie folgt auszuführen (Tab. 1):

**Table 1:** 

Datum:	Von:	Bis:
Bedingung A: mdd = *COPD*	01.01.2020	04.11.2020
Bedingung B: alt > 60J	01.01.2020	04.11.2020
Logische Verknüpfung: A + B		

*mdd* medizinische Daten, Karteizeilenkennung Diagnose, *alt* Alter

Im ersten Schritt fragten wir so die Gesamtzahl unserer COPD-Patient*innen im Alter über 60 Jahre ab. Dabei ergab sich eine Liste mit 45 Patient*innen. Bei ihrer Durchsicht stellten wir einige Probleme der initial noch zu einfach gehaltenen Listenabfrage fest. Die Liste enthielt z. B. auch Patient*innen, die bereits verstorben waren, die nicht mehr in unserer Praxis behandelt wurden sowie Patient*innen, bei denen die Diagnose „Ausschluss von COPD“ kodiert worden war. Die Konsequenz war, dass trotz der elektronischen Möglichkeit einer Listenerstellung dennoch die Karteikarten der Patient*innen auf der Liste einzeln gesichtet werden mussten, um die fehlerhaft in ihr enthaltenen Patient*innen herauszufiltern. Nach der Sichtung blieben 38 Patient*innen übrig, die unserem Indikatorrisikokollektiv der über 60-jährigen COPD-Patient*innen entsprachen.

Im zweiten Schritt ergänzten wir in der Abfrage den Impfstatus dieser Patient*innen (Tab. 2.):

**Table 2:** 

Datum:	Von:	Bis:
Bedingung A: mdd = *COPD*	01.01.2020	04.11.2020
Bedingung B: alt > 60J	01.01.2020	04.11.2020
Bedingung C: mdd = *Grippeimpfung*	01.10.2020	04.11.2020
Logische Verknüpfung: A + B + C		

*mdd* medizinische Daten, Karteizeilenkennung Diagnose, *alt* Alter

Eine solche Abfrage nach sog. Strings (Buchstabenketten, der Computer erkennt ja nicht den Sinn des Worts, sondern lediglich die Buchstabenfolge) ist nur zuverlässig möglich, wenn in unserem Fall die Grippeimpfung einheitlich als solche dokumentiert ist. Tippfehler oder die gelegentliche Dokumentation unter Influenzaimpfung würden unvermeidlich zur Nichterfassung einzelner Impfungen führen. Deshalb legen wir in unserem MVZ großen Wert auf einheitliche Dokumentation aus sog. Makros (kleine selbstzusammengestellte Dokumentationsbausteine, die nur noch bestätigt werden müssen) heraus.

Unter dieser Abfrage zeigten sich zunächst nur 2 Patient*innen (5 %), die den Kriterien entsprachen: Diagnoseeintrag COPD, über 60 Jahre, geimpft gegen Influenza im 4. Quartal 2020.

## Plan-do-check-act-Zyklus

Da das Ergebnis der ersten Abfrage eine Intervention zur Verbesserung der Impfrate mehr als sinnvoll erscheinen ließ, wurde ein „Plan-do-check-act“-Zyklus erstellt.

Der erste Schritt war das Anlegen und die konsekutive Führung einer Warteliste der impfwilligen Patient*innen. Impfwillige Patient*innen waren solche, die eigeninitiativ in der Praxis um einen Termin für eine Influenzaimpfung nachgefragt hatten. Innerhalb kurzer Zeit waren mehr als 200 Patient*innen auf der Warteliste und wir begannen mit der Priorisierung.

Unser PVS bzw. die Listenabfrage half bei der nachfolgenden breiteren Kategorisierung und Priorisierung der vulnerabelsten Patient*innen nicht weiter, mit deren Hilfe wir nicht mehr alleine Patient*innen mit COPD über 60 Jahren bevorzugen, sondern innerhalb der Warteliste der Impfwilligen auch Patient*innen mit (geplanter) Immunsuppression, aktiven malignen Erkrankungen und anderen chronischen Erkrankungen in die Priorisierung miteinschließen wollten. Eine Listenabfrage nach der kompletten Gruppe der Hochpriorisierten war aufgrund der sehr heterogenen Diagnosen in unserem PVS nicht möglich.

Die Kriterien dieser Kategorisierung wurden von uns nach bestem Wissen und in Anlehnung an die STIKO-Empfehlungen festgelegt [9]. Unsere Kriterien sind natürlich diskutierbar. Wir standen jedoch unter Zeitdruck und mussten die Kriterien schnell, praktikabel und im laufenden Betrieb festlegen. Die Einteilung erfolgte im Ampelsystem:

- Rot: Patient*innen über 60 Jahre und COPD, aktive maligne Erkrankung, (geplante) Immunsuppression;
- Gelb: Patient*innen über 60 Jahre mit chronischer Erkrankung (z. B. arterielle Hypertonie, Diabetes mellitus);
- Grün: alle weiteren Patient*innen.

Zur Kategorisierung war es notwendig, jeden einzelnen der Patient*innen auf der Warteliste im PVS aufzurufen und die Karteikarte mit Diagnosen und ggf. Medikation zu sichten. Diese Aufgabe wurde ärztlich durchgeführt. Besser geeignete PVS könnten diesen händischen Prozess deutlich erleichtern und beschleunigen.

In der Folge begann nun die strukturierte telefonische Einladung der als „Rot“ kategorisierten Impfwilligen, anschließend der „gelben“ Gruppe, zuletzt der „grünen“ ohne Risikofaktoren. Die Impfungen und weitere Kategorisierung erfolgten parallel im laufenden Prozess, da sich während dieser Phase auch weiterhin Patient*innen auf die Warteliste setzen ließen. Wir berichten hier jedoch im Sinne unseres Indikators exemplarisch nur die Ergebnisse für die COPD-Patient*innen.

Die zweite Abfrage erfolgte vier Wochen später. Erneut fragten wir nun exemplarisch unseren Indikator, die erreichte Impfquote der COPD-Patient*innen über 60 Jahre, als Teil unserer als „Rot“ kategorisierte Risikogruppe ab.

Die zu diesem Zeitpunkt noch nicht geimpften COPD-Patient*innen über 60 Jahre, die sich bis dahin noch nicht als impfwillig auf die Warteliste hatten setzen lassen, wurden einzeln von uns telefonisch kontaktiert, zur Influenzaimpfung eingeladen und wenn gewünscht geimpft.

## Ergebnisse

In der Abfrage nach Impfung aller Hochrisikopatient*innen auf der Liste der Impfwillen konnten wir feststellen, dass durch das o. g. Vorgehen die Impfrate unseres Indikators, der über 60-jährigen Patient*innen mit COPD, von 2 (5 %) auf 22 (58 %) der 38 Patient*innen gesteigert werden konnte.

Jedoch fiel uns zu diesem Zeitpunkt auf, dass Patient*innen, die in der hausarztzentrierten Versorgung (HZV) eingeschrieben waren, bei der Listenabfrage des Impfstatus nicht angezeigt wurden. Hierzu bedurfte es der zusätzlichen Abfrage mit einem eigenen Kürzel (mdd7 = *Grippeimpfung*). Um diese Abfrage ergänzt zeigte sich damit eine etwas höhere Impfquote zu Beginn von 3/38 (8 %) und eine Impfquote von 25/38 (66 %) nach Impfung der Risikogruppe auf der Liste der Impfwilligen.

Es entspricht vermutlich der Natur eines Plan-do-check-act-Zyklus, dass solche Fehler erst im laufenden Prozess auffallen und dann nachträglich korrigiert werden müssen. So stellt sich die Abfrage zusammen mit den HZV-Patient*innen im PVS wie folgt dar (Tab. 3.):

**Table 3:** 

Datum:	Von:	Bis:
Bedingung A: mdd = *COPD*	01.01.2020	03.12.2020
Bedingung B: alt > 60J	01.01.2020	03.12.2020
Bedingung C: mdd = *Grippeimpfung*	01.10.2020	03.12.2020
Bedingung D: mdd7 = *Grippeimpfung*	01.10.2020	03.12.2020
Logische Verknüpfung: A + B + (C/D)		

*mdd7* medizinische Daten D7, Diagnosen der HZV werden unter der Karteizeilenkennung D7 kodiert

Anschließend kontaktierten wir alle Patient*innen der Hochrisikogruppe, die sich nicht selbst auf die Liste der Impfwilligen hatten setzen lassen, telefonisch. Von den 13 noch ungeimpften COPD-Patient*innen unter ihnen konnten so nochmals drei Patient*innen mit der Impfung versorgt werden. Sechs der Patient*innen wünschten ausdrücklich keine Influenzaimpfung, ein Patient hatte sich bereits in einer anderen Praxis impfen lassen und 3 Patient*innen konnten telefonisch nicht erreicht werden. Als Gesamtergebnis nach Ende der Intervention und telefonischen Einladung waren abschließend 28 der 38 Patient*innen und damit 74 % der über 60-jährigen COPD-Patient*innen erfolgreich geimpft.

In unserer Liste der initial Impfwilligen konnte die gesamte Gruppe der als „Rot“ kategorisierten Patient*innen, (COPD, aktive maligne Erkrankung, [geplante] Immunsuppression) mit einer Influenzaimpfung versorgt werden.

## Diskussion

Durch die klinische Qualitätssicherungsmaßnahme waren 28 (74 %) der 38 Hochrisikopatient*innen nach unserer Intervention geimpft, sodass wir durch diese Maßnahmen den Zielwert der WHO von ca. 75 % Durchimpfung in der Interventionsgruppe der COPD-Patient*innen nahezu erreichten. Die PVS-Listenabfrage war hierbei hilfreich, auch wenn diese im Hinblick auf ihre Benutzerfreundlichkeit und Möglichkeiten deutlich verbessert werden könnte. Leider lässt sich aus unserer Erfahrung nur eine indirekte Anleitung für hausärztliche Kolleg*innen ableiten, da diese in der Mehrzahl andere PVS benutzen. In Deutschland gibt es 187 für Arztpraxen zugelassene PVS für verschiedene Anwendungsbereiche, sodass jede Kolleg*in mit ihrem eigenen PVS den konkreten Weg der KQS selbst finden muss. Dennoch haben wir unseren Abfragealgorithmus für das PVS MediStar hier und auf der Website unseres MVZ (https://www.mvz-eckental.uk-erlangen.de/klinische-qualitaetssteuerung/modellpraxis-mvz-eckental/download-und-feedbackbereich/) zur Verfügung gestellt (s. a. Infobox 1).

Sehr wichtig scheint uns, dass die Erhebung des Ist-Zustands zunächst so überraschend viel schlechter ausfiel als wir vermutet hatten. Es entspricht der hausärztlichen Realität, dass oft mit wenig Information gehandelt werden muss und die Prozesse erst im Verlauf optimiert und nachjustiert werden können. Es ist uns wichtig und wir sehen es als Teil unserer Professionalität an, diesen Prozess der KQS realistisch und daher mit seinen Tücken darzustellen und uns selbstkritisch zu prüfen [10]. Diese Erfahrung führte dazu, dass wir im Umgang mit der Listenabfrage unseres PVS sicherer geworden sind und die Abfragen heute präziser und schneller gestalten können. Dies wird in Zukunft dazu führen, dass wir auch für weitere Fragen der Versorgungsqualität, die im hausärztlichen Alltag auftauchen, mithilfe unseres PVS schnell Abfragelisten erstellen werden können und wollen. Die einmal durchgeführten Abfragen können gespeichert und so ohne großen Arbeitsaufwand z. B. in der Influenzaimpfsaison im nächsten Jahr wiederholt werden.

Unsere Vorerfahrung mit der Influenzaimpfung war im Verlauf der Pandemie dann sehr hilfreich im Umgang mit der Priorisierung und Verteilung der COVID-19-Impfungen.

### Diskussion anhand bestehender Literatur und bestehender elektronischer Impfmanagementsysteme

Es existiert ein guter Übersichtsartikel von Schelling et al. aus dem Bundesgesundheitsblatt 2019 zum Thema Impfmanagement [11]. Diese Arbeit beschäftigt sich jedoch, ähnlich wie andere Arbeiten [12], eher mit allgemeinen Fragen des Impfmanagements und nicht mit der Frage der Priorisierung und ihrer praktischen Umsetzung im Rahmen einer KQS. In der Arbeit von Schelling et al. wird eine Arbeit zitiert, die sich im deutschsprachigen Raum mit einer ähnlichen Fragestellung beschäftigt zu haben scheint, jedoch aktuell elektronisch nicht zugänglich ist [13]. Sie zeigt, dass sich mit dem elektronischen Impfmanagementsystem ImpfDoc deutliche Steigerungen von Impfraten erreichen lassen. Im Jahr 2012 waren jedoch nur 6000 von insgesamt etwa 55.000 deutschen Hausarztpraxen (11 %) im Besitz des Programms. Wie viele es davon wirklich nutzen, ist ungewiss. International gibt es eine Reihe von Arbeiten, die Verbesserungen der Impfquoten durch elektronische Erinnerungs- und Managementsysteme zeigen [14].

Die Schwierigkeit besteht jedoch darin, dass man in der Regel auf das eigene PVS angewiesen ist und sich innerhalb dessen kreativ Lösungen ausdenken muss. Es gibt allerdings zu den Basis-PVS hinzu buchbare elektronische Impfmanagementsysteme wie z. B. ImpfDocNE (https://www.impfdocne.de/) und Impfmodul (http://www.impfmodul.de/). Das Programm ImpfDocNE wurde vor Kurzem ganz neu entwickelt und scheint in der Lage, die von uns gewünschten Abfragelisten priorisierter Patient*innen zu erstellen und auch die Frage, wie viele von diesen bereits geimpft sind, beantworten zu können [15]. Auch andere elektronische Hilfen finden sich, wie z. B. https://www.impfterminmanagement.de/ und speziell für die Coronaimpfungen https://praxis.idana.com/de/ebook-impfmanagement. Das Problem aller elektronischen Impfmanagementsysteme ist, dass für die meisten Impfungen zunächst alle bisherigen Impfungen aus dem gelben Impfausweis in das Programm übertragen werden müssen. Bei der Influenzaimpfung, die ja jährlich verabreicht werden sollte, ist das freilich nicht nötig. Wir werden uns demnächst mit den Möglichkeiten von ImpfDocNE näher beschäftigen.

Auch gibt es andere PVS als das unsere, die die Aufgabe der Erstellung von Patient*innenlisten sehr viel besser zu ermöglichen scheinen als unser PVS MediStar. Als Beispiel sei hier das PVS EVA (abasoft) genannt, dessen Möglichkeiten in Kombination mit einem eigens entwickelten Datenimport- und Exportmodul kürzlich im Rahmen einer Forschungsarbeit zur Coronapandemie genutzt wurden [16].

Wir sehen unsere Methode der Listenabfrage, der Priorisierung und der telefonischen Erinnerung als einen möglichen Weg der KQS für alle Hausärzt*innen auch ohne besondere Informatikkenntnisse, wissenschaftlichen Hintergrund oder elektronische Zusatzprogramme an. Auch sind für andere klinische Fragestellungen kaum Programme vorhanden, mit denen sich eine KQS umsetzen ließe. Aufgrund der Erfahrungen und Schwierigkeiten mit den COVID-19-Impfungen stellen sich vermutlich viele hausärztliche Kolleg*innen die Frage, wie ein möglichst planvolles und gerechtes Vorgehen in der Hausarztpraxis aussehen könnte. Mit dieser Arbeit möchten wir einen Beitrag zur Problemlösung und Diskussion leisten und unsere Kolleg*innen dazu ermuntern, sich mit der Möglichkeit der Listenerstellung in ihren eigenen PVS auseinanderzusetzen und sie in den Praxisalltag zu integrieren.

Weitere Fragestellungen im Rahmen einer KQS könnten beispielsweise sein:

- Welche unserer Patient*innen mit schwerer Niereninsuffizienz erhalten Medikamente, die sie aufgrund dieser Diagnose nicht mehr erhalten sollten?
- Welche hochbetagten Patient*innen erhalten noch welche Medikamente, die z. B. auf der Priscus-Liste stehen?
- Bei wie vielen unserer Patient*innen mit Polypharmazie wurde im letzten Jahr der Medikamentenplan überprüft?
- Versorgen wir unsere Patient*innen mit Herzinsuffizienz leitliniengerecht?
- Ist die Indikation für verordnete Schleifendiuretika wirklich gegeben?
- Ist die Indikation für L‑Thyroxin-Verordnungen gegeben [17]?

## Schlussfolgerung

Auch wenn unser PVS, vermutlich ähnlich wie die meisten deutschen PVS, deutliche Defizite im Rahmen der Umsetzung einer KQS zeigte, war die Erstellung von Patient*innenlisten, eine Priorisierung der Patient*innen und die Organisation der Impfungen über diese Listen möglich. Vermutlich haben viele unserer Kolleg*innen, spätestens im Rahmen der COVID-19 Impfungen, bereits ähnliche Lösungen für eine systematische Herangehensweise bezüglich der eigenen Versorgungsqualität gesucht und ihre Erfahrungen gemacht. Wir wären dankbar, von diesen Erfahrungen zu hören, und sind gerne bereit, sie auf der Webseite unseres MVZ zu sammeln (s. Infobox 1). Dort ließe sich auch ein Erfahrungsaustausch und gegenseitige Hilfe für Nutzer der gleichen PVS organisieren. Unter der E‑Mail-Adresse in Infobox 1 können Sie uns Ihre Erfahrungen formlos mitteilen.

### Infobox 1 Erfahrungsaustausch

- Webseite des MVZ-Eckental, Universitätsklinikum Erlangen: https://www.mvz-eckental.uk-erlangen.de/klinischequalitaetssteuerung/modellpraxis-mvzeckental/download-undfeedbackbereich
- E-Mail: am-feedbackmodellpraxis@uk-erlangen.de
